# Interpretable Deep Learning Models for Arrhythmia Classification Based on ECG Signals Using PTB-X Dataset

**DOI:** 10.3390/diagnostics15151950

**Published:** 2025-08-04

**Authors:** Ahmed E. Mansour Atwa, El-Sayed Atlam, Ali Ahmed, Mohamed Ahmed Atwa, Elsaid Md. Abdelrahim, Ali I. Siam

**Affiliations:** 1 Electronics and Communication Department, College of Engineering and Computer Science, Mustaqbal University, Buraydah 51411, Saudi Arabia; 2 Department of Computer Science, College of Computer Science and Engineering, Taibah University, Yanbu 966144, Saudi Arabia; satlam@taibahu.edu.sa; 3 Computer Science Department, Faculty of Science, University of Tanta, Tanta 31527, Gharbia, Egypt; 4 Information Technology Department, Faculty of Computers and Information, Menoufia University, Shibin El Kom 6131567, Egypt; ali.ahmed@ci.menofia.edu.eg; 5 Faculty of Medicine Kasr Al-Ainy, Cairo University, Cairo 11562, Egypt; mo_atwa2022@students.kasralainy.edu.eg; 6 Computer Science Department, College of Science, Northern Border University, Arar 91431, Saudi Arabia; 7 Research Institute of Sciences and Engineering, University of Sharjah, Sharjah 27272, United Arab Emirates; ali.siam@sharjah.ac.ae; 8 Department of Embedded Network Systems Technology, Faculty of Artificial Intelligence, Kafrelsheikh University, Kafr El-Sheikh 33516, Egypt

**Keywords:** ECG signal analysis, arrhythmia detection, deep learning models, CNN, multiclass classification, biomedical signal processing

## Abstract

**Background/Objectives:** Automatic classification of ECG signal arrhythmias plays a vital role in early cardiovascular diagnostics by enabling prompt detection of life-threatening conditions. Manual ECG interpretation is labor-intensive and susceptible to errors, highlighting the demand for automated, scalable approaches. Deep learning (DL) methods are effective in ECG analysis due to their ability to learn complex patterns from raw signals. **Methods**: This study introduces two models: a custom convolutional neural network (CNN) with a dual-branch architecture for processing ECG signals and demographic data (e.g., age, gender), and a modified VGG16 model adapted for multi-branch input. Using the PTB-XL dataset, a widely adopted large-scale ECG database with over 20,000 recordings, the models were evaluated on binary, multiclass, and subclass classification tasks across 2, 5, 10, and 15 disease categories. Advanced preprocessing techniques, combined with demographic features, significantly enhanced performance. **Results**: The CNN model achieved up to 97.78% accuracy in binary classification and 79.7% in multiclass tasks, outperforming the VGG16 model (97.38% and 76.53%, respectively) and state-of-the-art benchmarks like CNN-LSTM and CNN entropy features. This study also emphasizes interpretability, providing lead-specific insights into ECG contributions to promote clinical transparency. **Conclusions**: These results confirm the models’ potential for accurate, explainable arrhythmia detection and their applicability in real-world healthcare diagnostics.

## 1. Introduction

The electrocardiogram (ECG) serves as a widely adopted, non-invasive diagnostic method for recording the heart’s electrical activity, offering crucial information about its rhythm and overall function. It plays a pivotal role in detecting a broad spectrum of cardiac abnormalities, particularly arrhythmias, irregular heart rhythms that can range from harmless to life-threatening, including conditions such as atrial fibrillation and ventricular tachycardia [1]. Timely detection and precise classification of these arrhythmias are vital for reducing the risk of severe outcomes such as stroke, heart failure, or sudden cardiac arrest. To support clinicians and improve diagnostic precision, automated ECG interpretation systems have been introduced. In this context, the PTB-XL dataset featuring over 20,000 labeled ECG recordings representing a wide range of cardiac conditions has emerged as a standard reference for assessing the performance of machine learning algorithms in arrhythmia classification [2].

Conventional ECG analysis techniques often depend on manual feature extraction and rule-based algorithms. Although these methods laid the groundwork for early developments in automated arrhythmia detection, they are constrained by their reliance on time-consuming procedures and their limited ability to capture the intricate and non-linear patterns present in ECG signals [3]. Traditional machine learning models, such as support vector machines (SVMs) and random forests, have been employed to mitigate some of these challenges [4]. However, they still require handcrafted features, which depend heavily on expert knowledge. In contrast, deep learning (DL) models offer a more advanced and efficient solution by automatically learning hierarchical representations directly from raw ECG data, bypassing the need for extensive preprocessing or manual intervention. Architectures like CNNs and recurrent neural networks (RNNs) have demonstrated superior performance in arrhythmia detection tasks, making them highly suitable for deployment in practical clinical settings [5].

Applying deep learning to ECG signal classification using the PTB-XL dataset presents several critical challenges. A major issue is class imbalance, where clinically important but infrequent arrhythmia are underrepresented, leading to biased model predictions that favor more common conditions. Additionally, ECG recordings are frequently contaminated with noise and artifacts, such as baseline drift and powerline interference, which can obscure relevant diagnostic features and negatively affect classification performance. The high dimensionality of multi-lead ECG signals further necessitates the use of complex architectures and demands significant computational resources. Generalizability also remains a concern, as models trained on PTB-XL may not perform consistently across different clinical environments due to variations in patient demographics, data acquisition protocols, and recording equipment. Finally, the “black-box” nature of deep learning models poses interpretability challenges, which may hinder clinician trust and limit their adoption in healthcare settings [2,6].

The critical importance of arrhythmia classification, coupled with the advantages and challenges of deep learning techniques, highlights the need for continued research in this domain [7]. Addressing these challenges and leveraging modern DL architectures can pave the way for robust, interpretable, and accurate arrhythmia detection systems, ultimately improving patient care and enabling seamless integration into clinical workflows [8].

The primary contributions of this study are as follows:

- A custom convolutional neural network-based deep learning model is proposed, specifically tailored for ECG arrhythmia classification. The model supports various diagnostic tasks, including binary classification (e.g., normal vs. specific conditions), and multiclass classification into 5, 10, or 15 clinically meaningful categories based on the PTB-XL dataset, allowing for both broad and detailed arrhythmia detection.
- To enhance contextual accuracy, the model integrates demographic attributes, namely age and gender, which contribute to a deeper understanding of arrhythmia patterns and lead to improved detection, particularly for less-represented classes.
- This research utilizes the PTB-XL dataset, a large and heterogeneous collection of ECG recordings, to ensure comprehensive training and validation. This choice supports the model’s generalizability across a broad range of cardiac conditions.
- An extensive experimental evaluation is conducted to assess the influence of incorporating demographic data. This study compares classification performance across binary and multiclass tasks, both with and without demographic features, demonstrating the added value of contextual inputs.
- In addition, the model offers interpretability by examining the influence of individual ECG leads in arrhythmia detection. This lead-specific analysis provides clinical insight into which signal channels most significantly contribute to the diagnostic process.

The remainder of this paper is organized as follows: Section 2 reviews the most significant and recent published works related to ECG-based arrhythmia classification. Section 3 describes the methodology employed in this study, including data processing, model architecture, and training procedures. Section 4 presents the experimental results in detail and highlights the advantages of the proposed approach compared with state-of-the-art methods. Section 5 concludes the paper by summarizing the main findings and outlining potential directions for future research.

## 2. Literature Review

The application of electrocardiogram (ECG) signals for arrhythmia classification has emerged as a prominent area of research due to its vital importance in the early diagnosis and prevention of cardiovascular diseases. Traditional methods largely dependent on manual feature engineering and classical statistical models are now being superseded by deep learning (DL) techniques, which facilitate automated feature extraction and significantly improve classification performance. Among the most influential resources in this domain is the PTB-XL dataset, a large-scale, richly annotated collection of ECG recordings that has become central to the development and benchmarking of DL-based arrhythmia detection systems. This study highlights recent advancements in the field, with particular focus on the challenges associated with leveraging deep learning models on the PTB-XL dataset.

A wide range of studies have employed various computational approaches for arrhythmia detection and classification, including traditional machine learning algorithms [9,10,11,12,13,14,15,16,17,18,19,20], transfer learning techniques [6,21,22,23,24] deep learning models [2,25,26,27,28,29,30,31,32], and hybrid frameworks that combine multiple methodologies [12,17,33,34,35,36,37,38,39,40]. Additionally, other innovative strategies have been explored in this context [41,42,43,44]. These efforts increasingly utilize large-scale ECG datasets such as PTB-XL and PTB, which offer diverse and clinically rich data for model development. Among the most influential resources is the PTB-XL dataset, which is extensively used in recent DL-based ECG research.

Recent progress in deep learning has significantly transformed ECG analysis, particularly through the use of large-scale datasets such as PTB-XL. A landmark study by Strodthoff et al. [2] provided a comprehensive benchmarking of deep learning architectures on the PTB-XL dataset, achieving macro-AUC scores of 0.93 for diagnostic categories and 0.96 for rhythm classifications using ResNet- and Inception-based networks. Their work also highlighted the advantages of transfer learning in improving ECG classification performance. Similarly, Jin et al. [5] proposed SJTU-ECGNet, a knowledge-integrated deep learning model trained on a large Chinese ECG dataset, which reported a mean accuracy of 93.74%, a macro-F1 score of 83.51%, and an AUC-ROC of 0.977.

To address challenges such as class imbalance and computational efficiency, a multi-receptive field convolutional neural network (MRF-CNN) was developed, achieving an F1 score of 0.72 and an AUC of 0.93 in superclass classification on PTB-XL [23]. In a different approach, Raymond Ao et al. explored image-based ECG classification by applying transfer learning with VGG16 on visual representations of 12-lead ECGs. Their model achieved an AUROC of 1.000 and a sensitivity of 0.992 for atrial fibrillation, demonstrating the potential of image-based techniques where raw signal data may not be available [25].

Hybrid deep learning models have also gained traction. Butt et al. [27] proposed a CNN-LSTM combination alongside a transformer model enhanced by wavelet transforms, achieving a remarkable 99.93% accuracy in detecting myocardial infarction. In another comparative study, Smigiel et al. [32] developed a CNN framework utilizing QRS complex segmentation along with entropy-based inputs, yielding an AUC of 96.3% for binary classification using PTB-XL data.

Transfer learning has been widely investigated in the context of ECG classification. In a comparative evaluation of six deep learning models, including ResNet1d18, it was shown that fine-tuning pretrained networks consistently outperformed models trained from scratch, with ResNet1d18 achieving an F1 score of 0.877 [43]. Jing et al. [45] introduced the beat-level fusion network (BLF-Net), which incorporates attention mechanisms to enhance feature learning. Their model achieved macro-AUC scores of 0.941 for subclass classification and 0.969 for rhythm detection, underscoring its potential for clinical application. Building upon this line of research, the PTB-XL+ dataset was introduced, offering feature-based annotations derived from both commercial and open-source algorithms. This enriched dataset supported improved model development and achieved macro-AUC scores as high as 0.889, providing a valuable foundation for advancing diagnostic tools [46].

Hambarde et al. [47] proposed the WOLF-DNN model, a hybrid deep neural network optimized using a wolf-inspired metaheuristic for arrhythmia detection from single-lead ECG signals. The model achieved an F1-score of 96.55% and sensitivity of 95.51%, demonstrating strong performance on imbalanced data. However, its generalizability remains limited due to the absence of testing on diverse, real-world clinical datasets. Further contributions by Strodthoff et al. [48] included the extraction of ECG features from commercial systems (Marquette 12SL and the University of Glasgow ECG program) and an open-source alternative (ECGDeli). Benchmark results indicated strong discriminative capability, with macro-AUCs of 0.889 for Uni-G, 0.871 for 12SL, and 0.879 for ECGDeli, reinforcing the effectiveness of feature-based approaches in ECG analysis.

Krasteva et al. [49] applied transfer learning using 18 pretrained ImageNet models on ECHOView images from Holter ECG recordings to detect atrial fibrillation. Their best fine-tuned models, such as EfficientNetV2B1, achieved up to 97.6% accuracy in binary classification. While the study demonstrates strong performance and model interpretability through GradCAM, it focuses solely on atrial fibrillation and relies on image-based input, limiting generalization to other arrhythmias or raw ECG data. Wickramasinghe and Athif [50] proposed an interpretable CNN model for multi-label classification of 26 cardiac abnormalities using reduced-lead ECGs. Trained on the PhysioNet 2021 dataset, their dual-branch model (time and frequency) achieved an F1 score of 0.553 on the hidden test set, ranking second for 12-lead, fifth for 6-lead, and third for 2-lead configurations. While SHAP improved interpretability, performance was limited by label imbalance and reduced-lead data constraints. Xiong et al. [4] proposed a 1D CNN model for classifying ECGs into normal, AF, other, and noise categories using the PhysioNet 2017 dataset. Their model achieved an F_1_ score of 0.82, outperforming RNN and spectrogram-based CNNs. The approach was limited to single-lead ECGs and showed lower performance on minority classes.

Recent systematic reviews have synthesized these advancements, providing an in-depth examination of current methodologies and their practical applications in ECG-based arrhythmia classification [51,52]. An overview of key studies utilizing this dataset is summarized in Table 1.

## 3. Methodology

**Figure 1:**
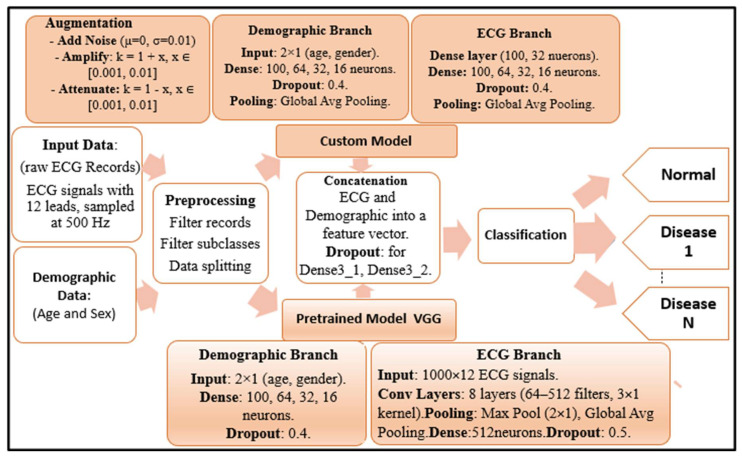
Overview of the proposed deep learning framework for arrhythmia classification.

The proposed hybrid deep learning framework for classifying ECG signals into normal and arrhythmic conditions is illustrated in Figure 1. The model integrates two distinct data streams: raw 12-lead ECG signals sampled at 500 Hz, and demographic information comprising patient age and sex. Prior to model training, the data undergoes a comprehensive preprocessing phase that involves filtering incomplete or noisy records, excluding underrepresented subclasses to ensure class balance, and splitting the dataset into training, validation, and testing sets.

To improve generalization and reduce overfitting, data augmentation techniques are applied exclusively to the ECG input branch. These augmentations simulate realistic physiological variations and include adding Gaussian noise with a mean of 0 and a standard deviation of 0.01, amplifying the signal by multiplying with a gain factor k = 1 + x where x ranges between 0.001 and 0.01, and attenuating the signal by applying a gain of k = 1 − x with the same range. These operations are designed to reflect natural fluctuations in ECG signals due to patient variability or sensor conditions.

The architecture is composed of two primary branches. The ECG branch is based on a convolutional neural network (CNN) and processes the augmented ECG signals through a stack of eight convolutional layers with 64 to 512 filters and 3 × 1 kernel sizes. Feature maps are reduced via max pooling and global average pooling. A dense layer with 512 neurons and a dropout rate of 0.5 is used for regularization. In parallel, the demographic branch processes the age and sex inputs through fully connected layers with 100, 64, 32, and 16 neurons, followed by global average pooling and a dropout of 0.4.

Following independent feature extraction, the outputs of both branches are concatenated to form a unified feature vector. This combined representation is passed through two additional dense layers (Dense3_1 and Dense3_2) with interleaved dropout layers (Dropout3_1 and Dropout3_2), both with a rate of 0.2, to prevent overfitting and enhance feature abstraction. The final vector is then passed to a classification layer with a softmax activation function to predict class probabilities for normal rhythm and various arrhythmic conditions (e.g., Disease 1 to Disease N).

The inclusion of demographic variables in the model architecture is motivated by clinical evidence linking age and gender to distinct arrhythmogenic patterns and ECG morphologies. Numerous cardiac conditions, such as myocardial infarctions (AMI, IMI), conduction blocks (e.g., LAFB/LPFB, CLBBB, CRBBB), hypertrophy (LVH), and ischemic abnormalities (ISCA, ISCI, ISC), demonstrate age- and gender-dependent variations in incidence, pathophysiology, and ECG presentation. For example, atrial fibrillation, conduction delays, and bundle branch blocks are significantly more prevalent in elderly patients due to age-related degenerative changes in the conduction system, such as fibrosis and calcification of the His–Purkinje network, which impair electrical propagation [55]. Gender differences also critically influence arrhythmia susceptibility and ECG morphology: women exhibit higher rates of long QT syndrome and non-specific ST-T changes, likely due to estrogen-mediated effects on ventricular repolarization [56], whereas men show greater prevalence of left ventricular hypertrophy (attributed to testosterone-driven myocardial remodeling) and Brugada-type patterns linked to sodium channel mutations [57,58]. By incorporating demographic features into the learning framework, the model is better equipped to contextualize ECG features and improve classification accuracy across diverse patient profiles.

### 3.1. Dataset Description

The PTB-XL dataset is a large-scale, publicly available resource designed to advance research in automated cardiac diagnostics. Developed and released by the Physikalisch-Technische Bundesanstalt (PTB), the dataset contains over 20,000 annotated 12-lead electrocardiogram (ECG) recordings from 18,885 patients, with a nearly balanced gender distribution (52% male, 48% female). As one of the most comprehensive ECG datasets available, PTB-XL offers greater diversity and volume than many existing alternatives. Each 10 s recording captures a wide range of cardiac conditions, annotated with 71 diagnostic labels systematically grouped into five hierarchical categories: diagnostic, form, rhythm, clinical, and additional statements.

Annotations were meticulously curated by two expert cardiologists and organized into superclasses and subclasses, providing a robust foundation for both supervised and unsupervised machine learning tasks. The dataset includes patients ranging in age from 18 to 95 years and spans both healthy individuals and those with various cardiac abnormalities, thereby enhancing its suitability for developing accurate and generalizable arrhythmia detection models.

Recordings are available at two sampling frequencies—100 Hz and 500 Hz—allowing researchers to adapt preprocessing based on their computational needs. Additionally, the dataset includes rich metadata such as patient age, gender, and recording conditions, which supports stratified analysis and the development of auxiliary learning tasks. In accordance with the recommendations of the PTB-XL dataset creators, we followed their predefined data split strategy:

- Folds 1–8 were used for training.
- Fold 9 was used for validation.
- Fold 10 was used for testing.

This split ensures consistency with prior studies and preserves the integrity of the evaluation [59]. Table 2 summarizes the dataset division.

### 3.2. Experimental Setup

The dataset employed in this study is organized using a predefined fold structure, enabling a standardized division into training, validation, and testing subsets for consistent evaluation of ECG classification algorithms. Specifically, the data is partitioned into 10 folds, with folds 1 through 8 allocated for training, fold 9 for validation, and fold 10 reserved for testing. This structured approach is critical for developing robust deep learning models while minimizing the risk of overfitting. Model training is guided by the cross-entropy loss function and optimized using the Adam optimizer with a learning rate of 0.001. To improve training performance and generalization, techniques such as early stopping and adaptive learning rate reduction are applied via callback functions. The experimental framework integrates both physiological signal data and demographic information, supporting accurate and reliable multiclass disease classification. All experiments were conducted on the Kaggle platform utilizing an NVIDIA TESLA P100 GPU accelerator.

### 3.3. Diagnostic Classes

**Figure 2:**
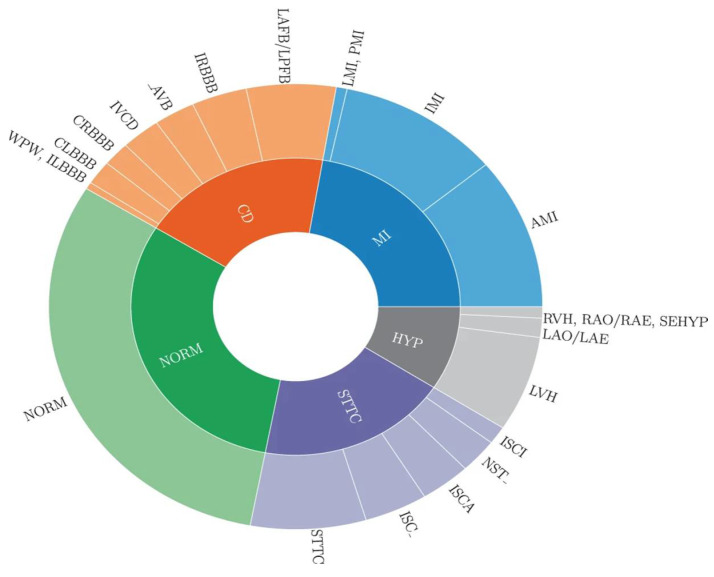
A summary of the PTB-XL dataset showing the diagnostic superclasses and subclasses.

This research focuses on developing an automated arrhythmia classification system based on ECG signals, capable of performing binary, multiclass, and subclass classification tasks. The PTB-XL dataset, used in this study, includes diagnostic labels categorized into five superclasses and 23 subclasses. Table 3 outlines the distribution of these superclasses and subclasses, while Figure 2 provides a visual representation of their relationships [46]. To evaluate the model’s performance comprehensively, we implemented multiple classification scenarios, including binary classification, 5-class (superclass), 10-class (subclass), and 15-class (subclass) tasks. These classification scenarios are described as follows:

A. Binary classification:

This scenario involves three cases:

Case 1: The model is designed to differentiate between normal ECG signals and one selected condition from the broader set of major disease classes, which includes CD, HYP, MI, and STTC.

Case 2: The model is employed to distinguish between normal ECG signals and one specific condition from a more detailed set of subclasses, such as STTC, AMI, IMI, LAFB/LPFB, LVH, IRBBB, CLBBB, ISCA, and CRBBB.

Case 3: The model is utilized to classify ECG signals as either normal or abnormal, with the abnormal category indicating the presence of a single underlying disease.

B. Multi-label classification

**Figure 3:**
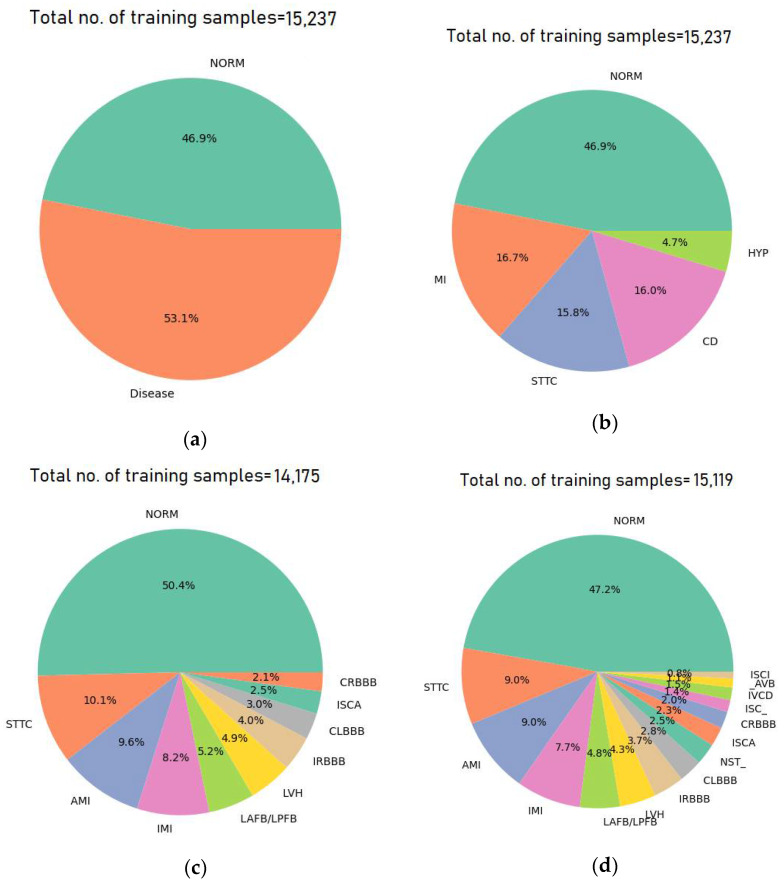
Class distribution before data augmentation for the training samples: (**a**) binary (normal and abnormal), (**b**) 5 superclasses, (**c**) 10 subclasses, and (**d**) 15 subclasses.

The class distributions for the binary, five-, ten-, and fifteen-class classification tasks, along with the number of records per class, are illustrated in Figure 3.

- Five-Class Supercategory Classification: In this multiclass classification task, the model categorizes ECG signals into one of five broad superclasses: NORM, MI, CD, STTC, and HYP.
- Ten-Class Subcategory Classification: In this configuration, the model classifies ECG signals into one of ten specific subclasses: NORM, STTC, AMI, IMI, LAFB/LPFB, LVH, IRBBB, CLBBB, ISCA, and CRBBB. These ten subclasses represent the most frequently occurring categories in the dataset, with all other less-represented subclasses being excluded.
- Fifteen-Class Subcategory Classification: This scenario expands the classification to fifteen subclasses, including: NORM, STTC, AMI, IMI, LAFB/LPFB, LVH, IRBBB, CLBBB, NST_, ISCA, CRBBB, IVCD, ISC_, _AVB, and ISCI. As with the ten-class configuration, only the top fifteen subclasses based on the number of records are considered.

### 3.4. Data Augmentation

**Figure 4:**
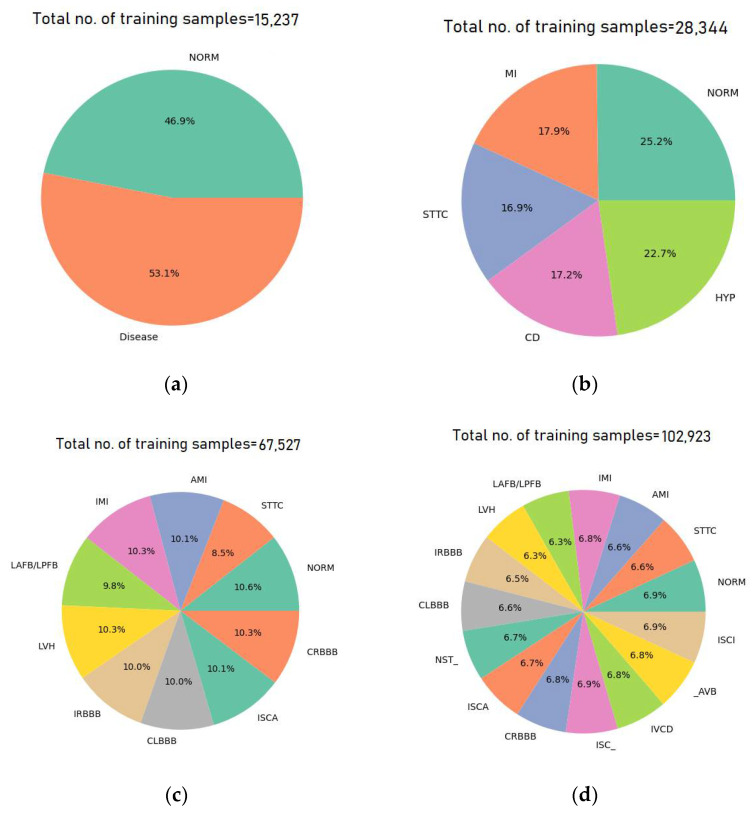
Class distribution after data augmentation for the training samples: (**a**) binary (normal and abnormal), (**b**) 5 superclasses, (**c**) 10 subclasses, and (**d**) 15 subclasses.

Data augmentation is crucial for improving the performance of deep learning (DL) models in classification tasks, especially when dealing with small datasets, as it artificially expands the training data by generating diverse and realistic variations (e.g., rotations, flips, scaling), helping to prevent overfitting and enhance generalization. For large datasets, data augmentation still plays a significant role by introducing additional variability that may not be fully captured, allowing the model to learn more robust and invariant features, ultimately leading to better accuracy and resilience to real-world data variations. In both cases, augmentation contributes to building more reliable and adaptable classification models. Table 4 shows the data augmentation techniques used in this study and the corresponding description. Figure 4 presents the class distribution of each category after data augmentation for the training samples.

### 3.5. The Proposed Models

#### 3.5.1. The Proposed CNN Model Architecture

**Figure 5:**
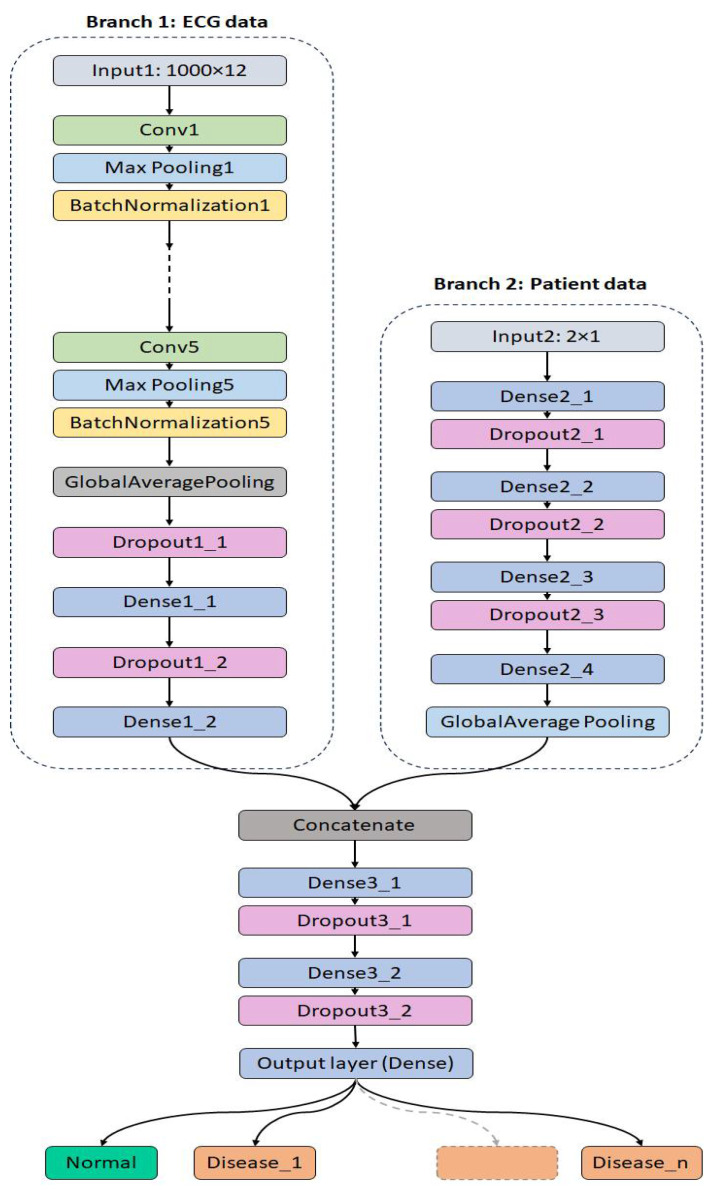
The proposed CNN model architecture.

The custom CNN architecture developed in this study adopts a dual-branch structure to independently process ECG signals and demographic data, as illustrated in Figure 5. The first branch is dedicated to handling raw ECG inputs and is composed of five sequential convolutional layers. These layers progressively increase in depth with filter sizes of 32, 64, 128, 256, and 512, respectively. Each convolutional layer utilizes a kernel size of 3 × 1, optimized for temporal feature extraction along the signal axis. Following each convolutional operation, a max pooling layer with a pool size of 2 × 1 is applied to downsample the feature maps and retain the most salient features. To promote training stability and accelerate convergence, each max pooling layer is immediately followed by a batch normalization layer. After the convolutional layers, the network transitions to two fully connected (dense) layers with 100 and 32 neurons, respectively. A dropout layer with a rate of 0.4 is employed to reduce overfitting by randomly deactivating neurons during training.

The second branch of the model processes demographic inputs (patient age and gender) through a series of four dense layers with 100, 64, 32, and 16 neurons. This branch also incorporates a dropout rate of 0.4, ensuring consistency in regularization across both branches. The output layer consists of a dense layer with a softmax activation function corresponding to the number of output classes (i.e., five for the ECG superclass classification). The architecture is designed to allow for effective feature learning from both physiological and contextual information. A full summary of the CNN model’s structural parameters is provided in Table 5.

#### 3.5.2. VGG Model Architecture

**Figure 6:**
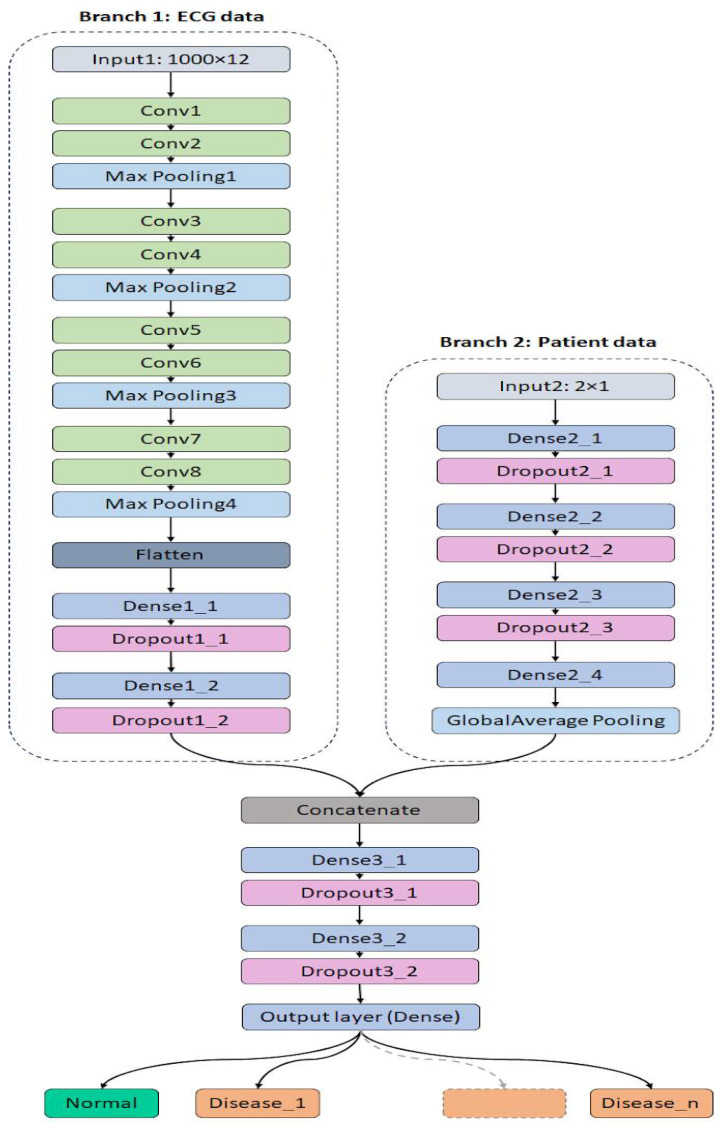
The proposed VGG model architecture.

The VGG-based model is an adaptation of the well-established VGG architecture, modified to support a multi-branch input structure for joint processing of ECG signals and demographic information. As illustrated in Figure 6, the ECG branch consists of eight convolutional layers, with filter sizes progressively increasing as follows: 64, 64, 128, 128, 256, 256, 512, and 512. Each convolutional layer employs a 3 × 1 kernel size, preserving temporal resolution while extracting relevant features. Following each convolutional block, a 2 × 1 max pooling operation is applied to downsample the spatial dimensions and facilitate hierarchical feature learning. This branch concludes with two fully connected layers, each containing 512 neurons, and incorporates a dropout rate of 0.5 to reduce overfitting and enhance generalization.

The demographic branch follows the same dense layer configuration used in the custom CNN model. It includes four fully connected layers with 100, 64, 32, and 16 neurons, respectively, along with a dropout rate of 0.4 for regularization. After parallel processing, the outputs from the ECG and demographic branches are concatenated and passed through a shared dense path composed of three layers with 10 neurons for each of the first two dense layers, and a final output layer, where the output dimension corresponds to the number of target classes (2, 5, 10, or 15, depending on the classification task). A dropout rate of 0.2 is applied in this final segment to ensure robustness.

The ReLU activation function is used throughout the model, except for the final classification layer, which uses softmax to output class probabilities. The model is trained using a categorical cross-entropy loss function and the Adam optimizer, with training hyperparameters and callbacks consistent with those used in the custom CNN model. Training is conducted over a maximum of 60 epochs, using a batch size of 16. A detailed summary of the model architecture and parameters is provided in Table 6.

### 3.6. Model Interpretability Using the SHAP Method

To improve the transparency and clinical relevance of the 12-lead CNN model, we employed SHAP (SHapley Additive exPlanations), a widely used model-agnostic interpretability framework [4]. SHAP quantifies the contribution of each input feature in this case, and each ECG leads to the model’s output. This allows us to assess how much each lead influences the model’s predictions for different cardiac conditions. For each arrhythmia class, SHAP values were computed across all test samples, and the absolute values were averaged to obtain a relative importance score for each lead. This approach provides an interpretable measure of the lead-specific contributions to the model’s decision-making process, aligning with clinical expectations and aiding in the trust and validation of AI predictions [50]. The relative importance $I_{l}\;$of lead l is calculated as:

(1) $$I_{l}=\frac{1}{N}\sum_{i=1}^{N}\left|{SHAP}_{il}\right|$$

where *N* is the number of test samples, and ${SHAP}_{il}$ is the *SHAP* value for lead l in sample *i*.

### 3.7. Performance Evaluation Metrics

To evaluate the performance of machine learning models in ECG arrhythmia classification, a set of key performance metrics is employed, including precision, recall, accuracy, and the confusion matrix. These metrics offer a comprehensive understanding of the model’s effectiveness, especially in the context of imbalanced datasets, where relying solely on accuracy can be misleading [59,60].

Accuracy reflects the overall correctness of the model by measuring the proportion of true predictions both for normal and arrhythmic cases, relative to the total number of predictions. It is mathematically defined as:

(2) $$Accuracy=\frac{TP+TN}{TP+TN+FP+FN}$$

In this context, *TP* refers to true positive instances where the model correctly identifies the presence of a disease. *TN* denotes true negatives, indicating correctly classified normal instances. *FP*, or false positives, occur when normal instances are incorrectly classified as diseased, while *FN*, or false negatives, represent diseased cases mistakenly labeled as normal. Although accuracy provides a general assessment of model performance, it can be misleading in cases of class imbalance, where the majority class disproportionately influences the metric.

To address this limitation, precision, also known as the positive predictive value, is used to evaluate the correctness of positive predictions. It quantifies the proportion of true positives among all instances that the model predicted as positive and is defined as:

(3) $$Precision=\frac{TP}{TP+FP}$$

This metric is particularly important in contexts where false positives carry serious consequences, as in medical diagnostics, where misclassifying a healthy individual as diseased can lead to unnecessary tests, treatments, and patient anxiety. In such cases, maintaining a high precision is essential to ensure that positive predictions are as reliable as possible.

On the other hand, recall, also referred to as sensitivity or the true positive rate, measures the model’s ability to correctly identify actual positive cases. It represents the proportion of true positives captured out of all real instances of the condition and is especially important when the cost of missing a positive case (i.e., a false negative) is high. Mathematically, recall is defined as:

(4) $$Recall=\frac{TP}{TP+FN}$$

High recall is particularly crucial in situations where overlooking true positive cases i.e., false negatives, can have severe consequences, such as the failure to detect life-threatening arrhythmias. In these scenarios, prioritizing sensitivity ensures that most relevant cases are correctly identified, even if it comes at the cost of a higher false positive rate. To further analyze model performance, the confusion matrix offers a comprehensive summary of predictions across all classes. It organizes the results into four key components: true positives (*TPs*), true negatives (*TNs*), false positives (*FPs*), and false negatives (*FNs*). This matrix provides valuable insight into how the model performs for each class, highlighting both its strengths and areas requiring improvement. Table 7 presents the structure and components of a confusion matrix, detailing how predictions are distributed across correct and incorrect classifications.

## 4. Experimental Results and Discussion

This section provides a detailed evaluation of the proposed deep learning frameworks for ECG signal arrhythmia classification, utilizing the PTB-XL dataset. The experiments assess model performance across various classification tasks, including binary classification, multiclass classification involving 5 superclasses, and subclass classification with 10 and 15 categories. The study emphasizes the integration of patient demographic data, such as age and gender, alongside raw ECG signals to enhance diagnostic accuracy, particularly for underrepresented arrhythmia classes. Comparative analyses with existing state-of-the-art methods highlight the superiority of the proposed approach.

The results of the study on ECG signal arrhythmia classification using deep learning techniques are categorized into various classification tasks, including binary classification and multiclass classification, with and without incorporating patient data. These findings highlight the impact of patient data inclusion on model performance, measured through metrics such as precision, recall, and accuracy.

### 4.1. Experimental Results Using the CNN Model

**Figure 7:**
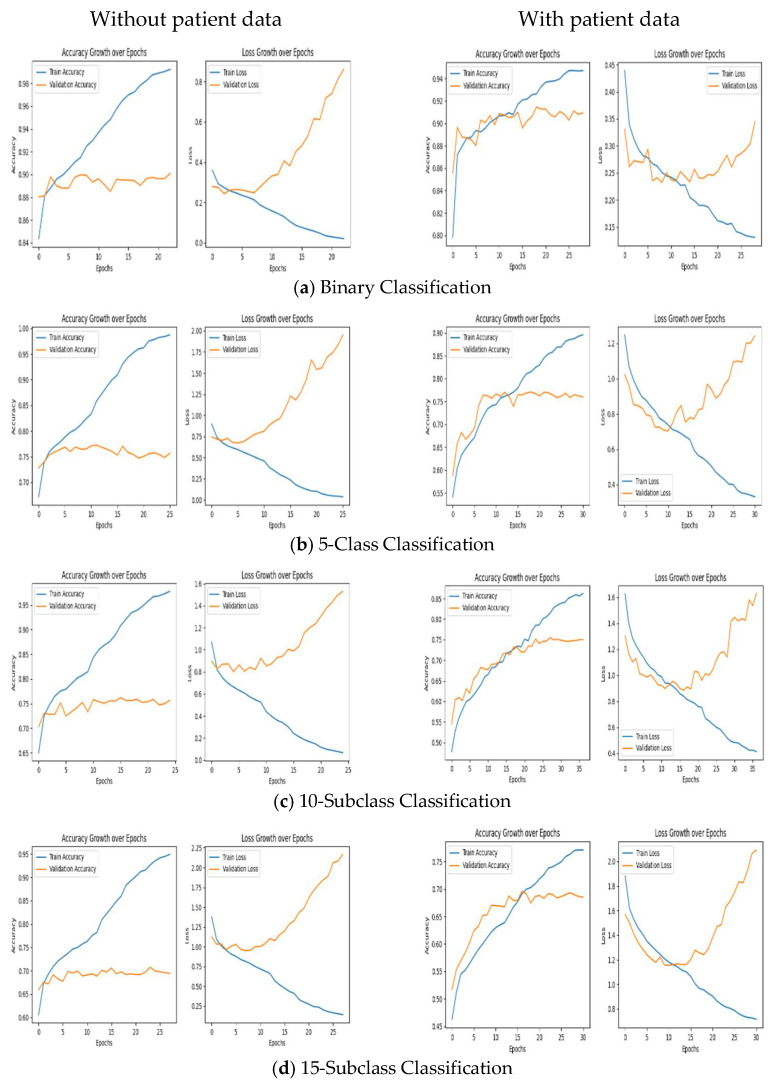
CNN training/validation accuracies and losses for all classes.

The training and validation performance of the CNN model was evaluated across all classification tasks, including binary and multiclass classifications. As shown in Figure 7, training and validation accuracies exhibited a consistent upward trend during model training, reflecting the effective learning of features from the PTB-XL dataset. For binary classification, the model achieved high accuracies across all subclasses, indicating robust generalization. Similarly, in multiclass classification, the training accuracy progressively improved while validation accuracy remained closely aligned, highlighting the model’s ability to handle diverse arrhythmia classes. Training and validation losses, monitored throughout the learning process, decreased steadily until reaching a specific epoch corresponding to the minimum loss value. Beyond this point, the losses began to increase, indicating the onset of overfitting. To address this, the parameter values corresponding to the epoch with the minimum loss were selected, ensuring the model’s optimal performance before overfitting occurred. These trends were consistent across experiments with and without patient data.

**Figure 8:**
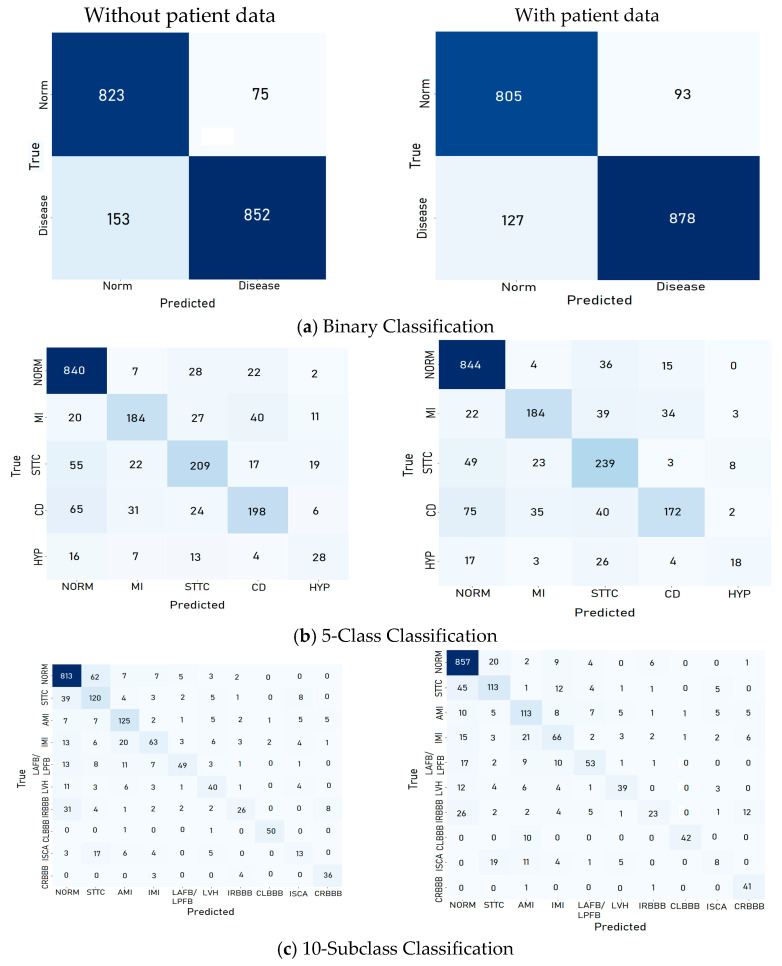

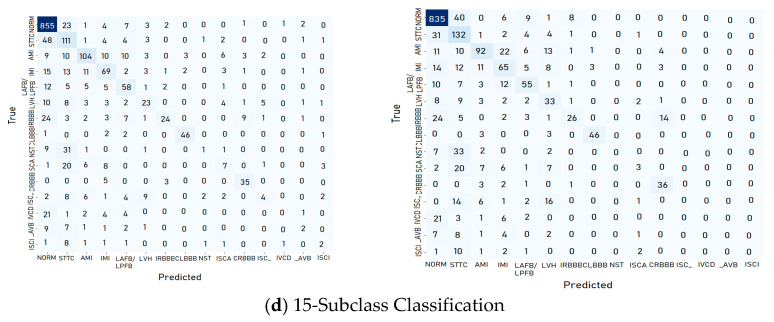
Confusion matrix for each category using the CNN model for the test set.

The confusion matrices generated for each classification category for the test set provide detailed insights into the performance of the proposed CNN model. For binary classification tasks, the matrices illustrate a high number of true positive (TP) and true negative (TN) predictions, reflecting the model’s ability to accurately distinguish between normal and abnormal ECG signals, as shown in Figure 8. Misclassifications, represented by false positives (FPs) and false negatives (FNs), were minimal, particularly when patient data was incorporated, underscoring the value of demographic information in improving classification accuracy.

#### 4.1.1. Binary Classification Test Results

**Figure 9:**
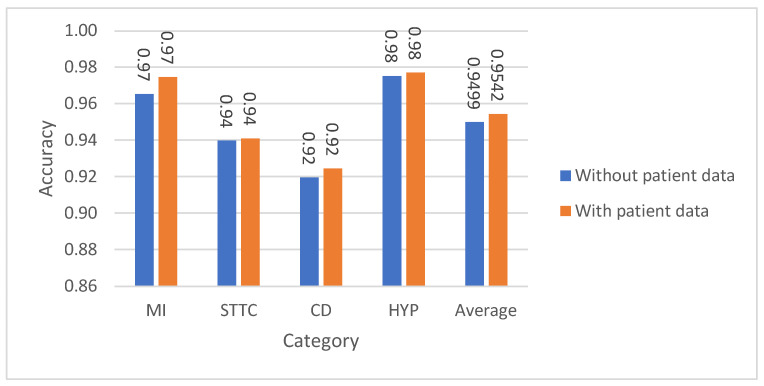
The classification results for the binary classification (normal vs. one of the remaining four superclasses) with and without patient data using CNN for the test set.

**Figure 10:**
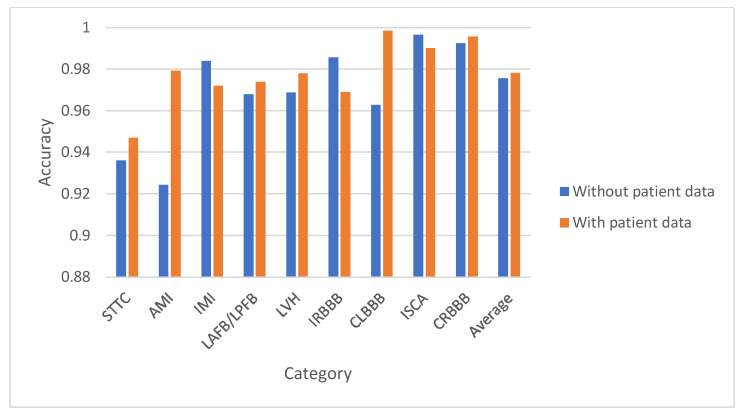
The classification results for the binary classification (normal vs. one of the remaining 9 subclasses) with and without patient data using CNN for the test set.

The accuracy results for binary classification on the test set, presented in Table 8, Table 9, Table 10 and Table 11, demonstrate strong performance of the CNN model across different binary classification scenarios. In Table 8 and Figure 9, distinguishing between normal and individual superclasses yielded accuracy ranging between 91.95% and 97.52%, with an average of 94.99% without patient data, which improved to 95.42% with demographic information. Table 9 and Figure 10 highlight subclass classification, where average accuracy reached 97.56%, and Table 10 shows further enhancement to 97.78% when patient data was included. Table 11 covers binary classification of normal vs. abnormal ECG signals, reporting 88.21% accuracy without patient data and 89.34% with it. Overall, the CNN model displayed excellent discriminative ability, especially when enhanced with demographic inputs, showcasing its robustness in binary ECG arrhythmia classification.

#### 4.1.2. Multiclass Classification Results

Multiclass classification results on the test set using the CNN model, both with and without patient demographic data, are presented in Table 12. The CNN model achieved an accuracy of 79.49% for five superclasses, 78.35% for 10 subclasses, and 73.70% for 15 subclasses. When patient data was incorporated, the accuracy slightly improved to 79.55%, 79.60%, and 72.61% respectively. These results highlight that integrating demographic information (age and gender) consistently enhances classification accuracy. The drop in accuracy for the 15-class case underscores the increased complexity and class imbalance challenges associated with fine-grained classification.

In multiclass and subclass classifications, the confusion matrices revealed a strong diagonal dominance, indicating accurate predictions across most arrhythmia categories. However, some overlap was observed in subclasses with similar ECG characteristics, such as STTC and CD. These findings highlight areas for improvement, particularly in reducing false negatives for underrepresented classes. Overall, the confusion matrices validate the model’s high precision and recall across all categories, demonstrating its reliability in clinical and diagnostic applications.

### 4.2. Experimental Results Using the VGG16 Model

The binary classification accuracy results using the VGG16 model on the test set, as presented in Table 13, Table 14 and Table 15, show strong performance across different diagnostic scenarios. For binary classification between “normal” and one disease from the remaining four subclasses (MI, STTC, CD, and HYP) with patient data, the model achieved accuracy values ranging from 92.02% to 97.52%, with an average of 94.54%. When distinguishing between “normal” and individual subclasses (such as AMI, CLBBB, and CRBBB), accuracy further improved, reaching as high as 99.94%, with an average accuracy of 97.38%. However, in the more generalized binary classification task of “normal” vs. “abnormal” ECG signals, the model’s performance dropped slightly to 87.29%. These results highlight VGG16′s effectiveness in fine-grained binary classification, though its performance is relatively modest in broader binary classification tasks.

Table 16 presents the multiclass classification accuracy results on the test set using the VGG16 model across increasing classification complexity (i.e., 5 superclasses, 10 subclasses, and 15 subclasses). The model achieved an accuracy of 76.53% for classifying ECG signals into 5 superclasses, slightly improving to 76.85% for 10 subclasses, which suggests a relatively stable performance despite the added complexity. However, the accuracy declined to 69.29% for 15 subclasses, highlighting the challenges of data imbalance and increased class overlap as the classification granularity intensifies. These results reflect the VGG16 model’s moderate capability in handling multiclass ECG classification, with performance gradually decreasing as the number of classes increases.

### 4.3. Performance Evaluation of CNN Against VGG16

**Figure 11:**
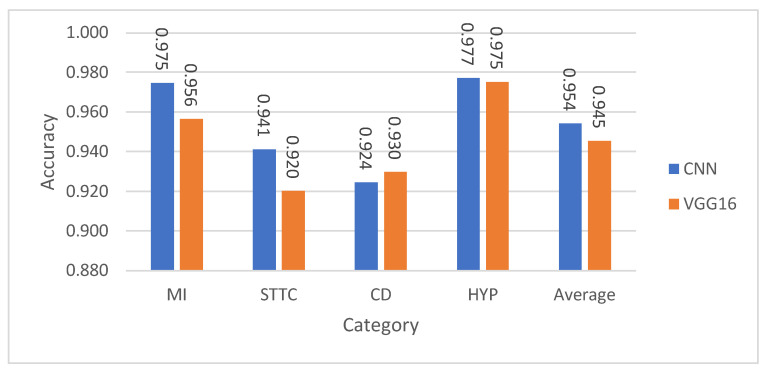
Performance comparison of CNN against VGG16 in the 5-superclass binary classification for the test set.

**Figure 12:**
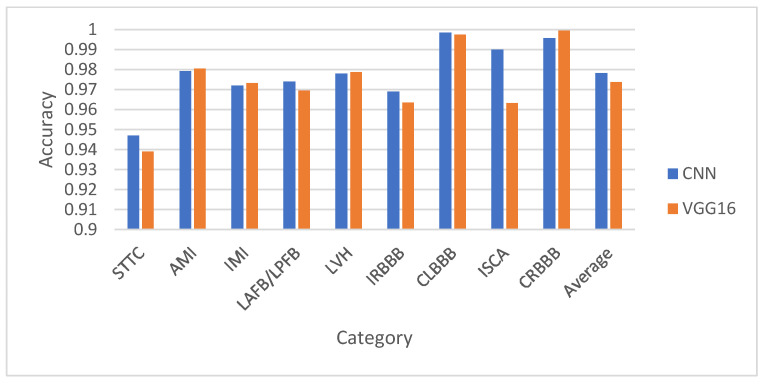
Performance comparison of CNN against VGG16 in the 10-subclass binary classification for the test set.

**Figure 13:**
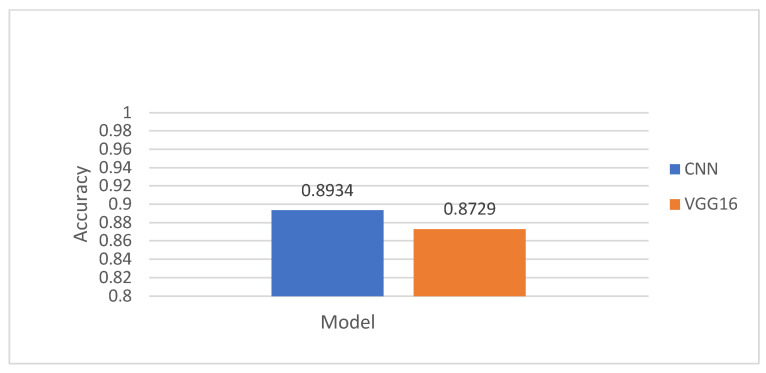
Performance comparison of CNN against VGG16 in the normal and abnormal binary classification.

**Figure 14:**
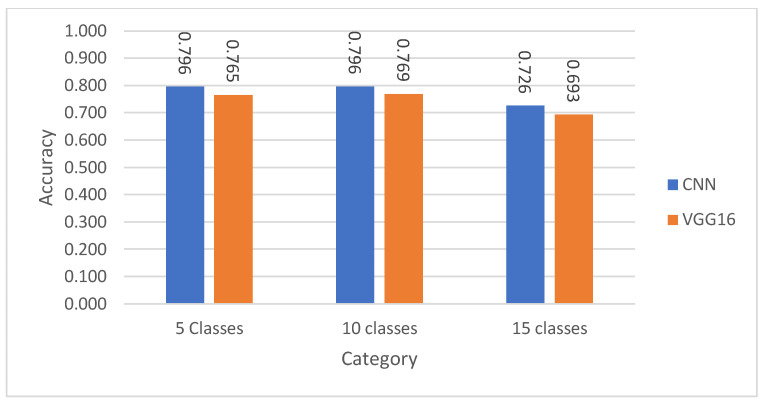
Performance comparison of CNN against VGG16 in the multiclass classification.

This section presents a comparative analysis of the proposed CNN model and the VGG16 architecture across various binary and multiclass ECG classification tasks. Figure 11, Figure 12, Figure 13 and Figure 14 show the classification accuracy comparisons between the custom CNN model and the VGG16 model. Overall, the CNN model consistently outperforms VGG16 in both binary and multiclass classifications. In binary classification, as demonstrated in Figure 11, CNN shows higher accuracy than VGG16 for all five superclasses. For the 10-subclass binary classification as declared in Figure 12, CNN leads in most cases, though VGG16 slightly outperforms it in specific subclasses like STTC, IMI, and CLBBB. In the binary classification of normal vs. abnormal ECG signals (Figure 13), CNN again demonstrates superior accuracy. Finally, in multiclass classification, covering 5 superclasses, 10 subclasses, and 15 subclasses, the CNN model outperforms VGG16 in all scenarios, as shown in Figure 14. These results highlight CNN’s greater robustness and generalization ability in handling both simple and complex classification tasks.

### 4.4. Performance Comparison with Recently Published Works

The performance evaluations of the proposed models are evaluated against some recently published works. In Table 17, the proposed models, CNN and VGG16, are compared with recently published works [27,31,32,44,54,61] for binary and multiclass classifications. The proposed CNN model, incorporating patient data, achieved the best results in binary classification (89.34%, 95.42%, and 97.78%), outperforming the others. In case of multiclass classification, CNN achieved an accuracy of 79.55%, 79.60%, 72.61% at 5 classes, 10 classes, and 15 classes, which was slightly better than VGG16′s 76.53%, 76.85% and 69.29%, respectively. These results underscore the superior performance of the proposed models, especially when patient demographic data is included, demonstrating their robustness in complex classification tasks.

### 4.5. Augmentation-Based Model Results

**Figure 15:**
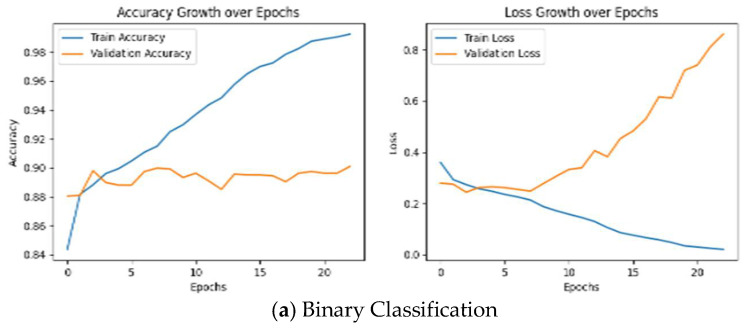

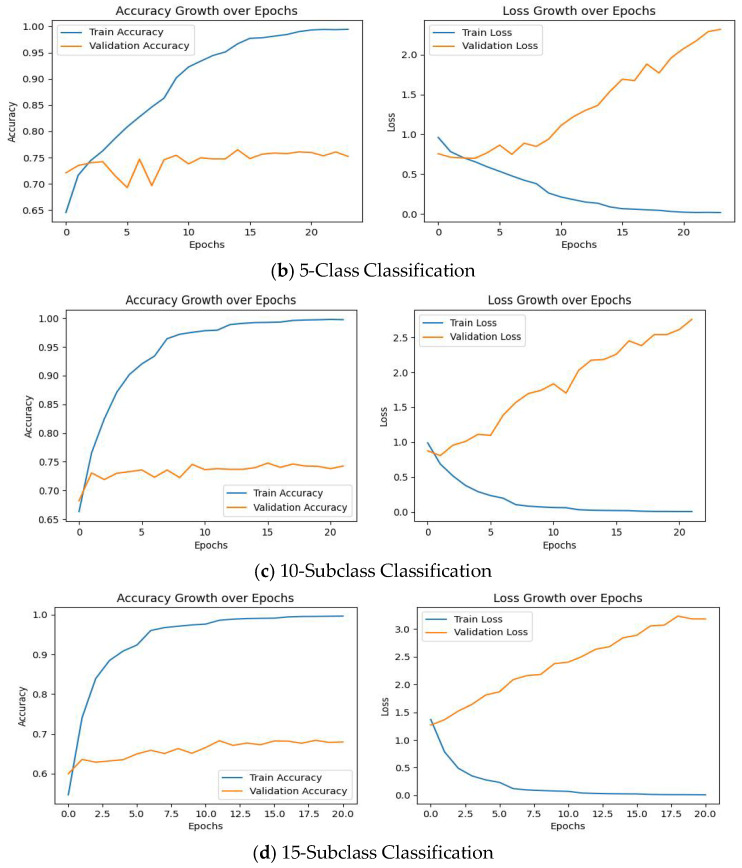
CNN training and validation accuracies for all classes as well as training and validation losses.

**Figure 16:**
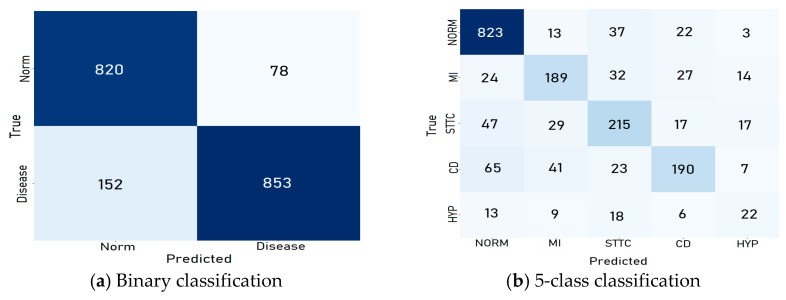

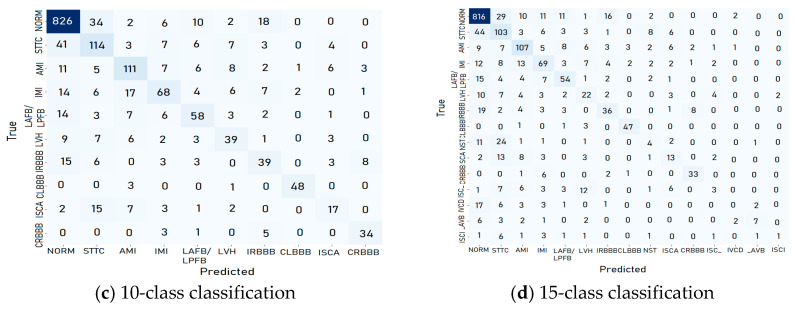
CNN confusion matrices for each category for the test set.

This section presents the performance of the CNN model when trained with augmented ECG data. To assess the impact of augmentation techniques such as noise addition, amplification, and attenuation on classification accuracy and robustness, we evaluate both binary and multiclass classification tasks. Figure 15 shows the training and validation accuracies for all classes as well as training and validation losses using the CNN model. In addition, Figure 16 presents confusion matrices for different ECG arrhythmia classification categories, offering visual insight into the classification accuracy of the CNN model across binary and multiclass scenarios.

Based on the results presented in Table 18, Table 19, Table 20 and Table 21, the use of augmented ECG data significantly enhanced the performance of the CNN model across all binary and multiclass classification tasks. In the binary classification of normal versus one disease from the remaining four superclasses, the model achieved an average accuracy of 94.28% with particularly high precision and recall values across all classes, as shown in Table 18. Also, in the binary classification involving 10 subclasses, the model showed exceptional accuracy, averaging 97.68%, with some disease classes such as CLBBB and CRBBB achieving a perfect classification of 100% accuracy, as presented in Table 19. In addition, Table 20 shows the binary classification of normal versus abnormal results; the model maintained a solid accuracy of 88.93%. Meanwhile, in multiclass classification tasks, the model achieved accuracies of 76.89%, 78.89%, and 71.47% for 5, 10, and 15 subclasses, respectively, as declared in Table 21. These findings indicate that data augmentation effectively boosts classification accuracy and model robustness, particularly in fine-grained classification scenarios involving multiple arrhythmia subclasses.

The accuracy results of the CNN model reveal the positive impact of data augmentation on ECG arrhythmia classification performance. Without data augmentation, the model achieved an average accuracy of 95.42% for binary classification (normal vs. superclasses) and 97.78% for normal vs. subclass scenarios. Upon applying data augmentation techniques such as noise addition, amplification, and attenuation, the model maintained high performance, with average accuracies of 94.28% and 97.68% for the same classification tasks, respectively. In the binary classification of normal vs. abnormal ECGs, the accuracy slightly decreased from 89.34% to 88.93% when applying data augmentation. Table 22 presents the performance comparison of CNN models on ECG signal classification, with and without data augmentation. The results show that the CNN model achieved accuracy when applying data augmentation slightly below the accuracy results without applying data augmentation. Although data augmentation is widely used to improve model generalization, the modest decrease in accuracy observed here suggests that simple additive noise, amplification, and attenuation may not be sufficiently representative of real-world ECG variations. Given the high fidelity of the PTB-XL dataset and the CNN’s strong baseline performance, these augmentations likely introduced distortions that interfered with subtle diagnostic features rather than enhancing the model’s robustness. Future work could explore domain-specific augmentations (e.g., lead dropout simulation, heart rate variability, or realistic arrhythmia patterns) to better mimic clinical conditions and improve performance.

### 4.6. Explainable ECG Channel Contributions for Arrhythmia Detection

**Figure 17:**
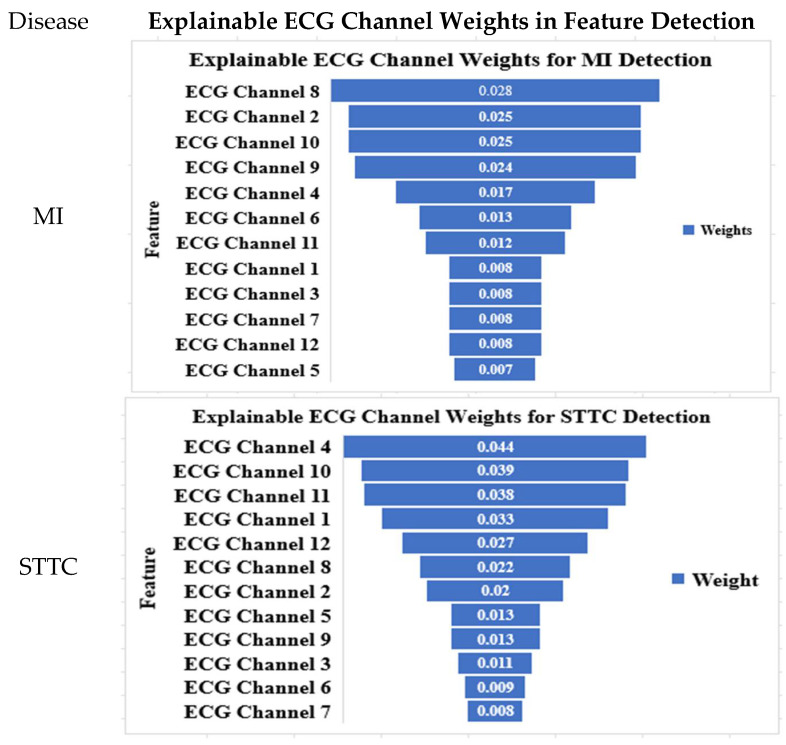

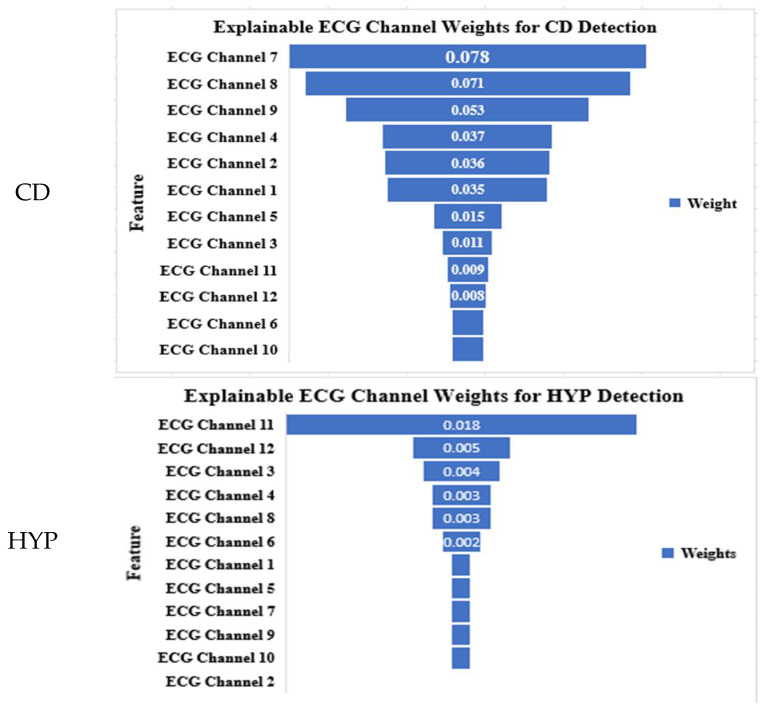
Explainable ECG channel weights for feature detection in binary classification (normal vs. one disease) based on the CNN model.

Figure 17 illustrates the CNN model’s lead-specific weight distribution, offering a clear view into how it prioritizes ECG channels for arrhythmia detection. This visualization enhances transparency and reflects alignment with clinical standards. For instance, leads V2, II, V3, and V4 were emphasized in detecting myocardial infarction (MI), mirroring the clinical focus on precordial and inferior leads. In cases of ST-T changes (STTCs), leads V4, V5, and aVR stood out, with aVR’s prominence corroborating its link to left main coronary artery disease [61]. For conduction disorders, leads V1–V3 were dominant, supporting right bundle branch block (RBBB) diagnosis [63], while the underrepresentation of lateral leads in left bundle branch block (LBBB) detection likely reflects dataset imbalance [46]. Hypertrophy detection aligned with voltage-based criteria (e.g., Sokolow–Lyon index) through leads V5 and V6 [64]. These patterns validate traditional workflows while offering novel risk stratification insights. The model’s emphasis on aVR, a lead often overlooked, clinically suggests the potential for the early identification of high-risk ischemia [65]. Prioritizing leads like V2–V4 for MI detection could streamline ECG analysis in resource-limited or emergency settings.

By mapping AI-derived lead importance to clinical indicators [66], the framework in the figure bridges AI diagnostics with medical reasoning. Clinicians gain tools to audit decisions, fostering trust in automation, while adaptive applications (e.g., wearables with condition-specific lead sensitivity) become feasible [67]. This integration of explainable AI enhances diagnostic accuracy, workflow efficiency, and data-driven care delivery.

## 5. Conclusions

This study proposed two explainable deep learning frameworks, CNN and VGG16 models, for ECG signal arrhythmia classification using the PTB-XL dataset, demonstrating their effectiveness across binary and multiclass classification tasks. By incorporating patient demographic data such as age and gender, the models achieved notable improvements in diagnostic accuracy, particularly in distinguishing complex or underrepresented arrhythmia subclasses. A key contribution of this work lies in its integration of explainable AI, which provided transparent insights into ECG channel importance across different cardiac conditions, reinforcing clinical relevance and trust in automated systems. The use of explainability not only enhanced the interpretability of predictions but also aligned model focus with established diagnostic practices, facilitating potential real-world adoption in healthcare. These findings affirm the critical role of explainable deep learning in developing accurate, reliable, and clinically meaningful ECG analysis systems and underline the importance of continued research in this domain. Future work will explore transformer-based models to better capture temporal dependencies in ECG signals. Integrating handcrafted features with deep learning may enhance classification accuracy and robustness. Cross-dataset evaluation is also important to assess generalizability across different clinical settings. Additionally, improving model interpretability through attention mechanisms or relevance-based methods will support clinical trust and real-world deployment.

## Figures and Tables

**Table 1:** Summary of research studies utilizing the PTB-XL dataset for arrhythmia detection and classification.

Ref.	Dataset	Models Applied	Key Results	DOI/Link
[2]	PTB-XL, ICBEB2018	ResNet, Inception, Transfer Learning, xresnet1d101	Demonstrated PTB-XL as a benchmark for ECG analysis; transfer learning showed promising results for small datasets.	https://doi.org/10.1109/JBHI.2020.3022989
[5]	Chinese ECG Benchmark	Knowledge-Fused DNN	Achieved higher performance than cardiologists on arrhythmia classification in remote settings.	https://doi.org/10.1038/s43856-024-00464-4
[23]	PTB-XL, CPSC2018, Georgia, Ribeiro	Transfer Learning (Various CNNs, RNNs)	Transfer learning was effective on small datasets; fine-tuning did not consistently outperform training from scratch on larger dataset.	https://doi.org/10.48550/arXiv.2402.02021
[25]	PTB-XL, CPSC2018, Shaoxing, Tongji	Convolutional Neural Networks (CNNs)	Excellent AUROC, AUPRC on test data from PTB-XL; lower performance on unseen datasets].	https://doi.org/10.1101/2022.11.21.22282586
[27]	PTB-XL	CNN-LSTM, Attention Transformer	Achieved 91.07% accuracy (MI detection)	https://doi.org/10.1109/ACCESS.2022.3220670
[31]	PTB-XL	1D CNN, LSTM, GRU, Multimodal Fusion, Attention-based models	GRU achieved 79.67% sensitivity and 81.04% specificity. The 1D representation outperformed 2D representations	https://doi.org/10.1016/j.bspc.2024.106141
[32]	PTB-XL	CNN, QRS complex extraction, Entropy-Based Features	Improved performance by adding entropy-based features to raw signals.	https://doi.org/10.3390/s21248174
[41]	PTB-XL	Beat-Level Fusion Network (BLF-Net)	Outperformed state-of-the-art methods in multiclass arrhythmia classification.	https://doi.org/10.1155/2023/1755121
[43]	PTB-XL, various smaller datasets	MELEP, CNNs, RNNs	MELEP effectively predicted transferability; strong correlation with fine-tuning performance (0.6+ correlation)	https://doi.org/10.1007/s41666-024-00168-3
[44]	PTB-XL	Few-Shot Learning (FSL), CNN	FSL achieved 93.2% accuracy for 2-class classification, outperforming softmax-based models.	https://doi.org/10.3390/s22030904
[45]	PTB-XL	AlexNet, LeNet	AlexNet performed better with high classification accuracy for cardiac conditions	https://www.researchgate.net/publication/374061560 accessed on 21 June 2025
[48]	PTB-XL	ECGDeli, Marquette 12SL	Introduced ECGDeli, Marquette 12SL feature sets for enhanced ECG interpretation.	https://doi.org/10.1038/s41597-023-02153-8
[53]	PTB-XL	Multi-Receptive Field CNN (MRF-CNN)	Achieved 0.72 F1 score, 0.93 AUC for 5 superclasses on PTB-XL dataset	https://doi.org/10.1155/2022/8413294
[54]	PTB-XL	CNN, SincNet, Entropy-Based Features	Best performance with convolutional network + entropy features.	https://doi.org/10.3390/e23091121

**Table 2:** PTB-XL dataset splitting into training, validation, and testing.

Subset	Folds	Size
Train	1–8	15,237
Validation	9	1886
Test	10	1903

**Table 3:** The superclasses and the subclasses in the PTB-XL dataset.

Superclass	Description	Subclass	Description
NORM.	Normal ECG	NORM	Normal ECG
CD	Conduction Disturbance	LAFB/LPFB	Left anterior/Left posterior fascicular block
		IRBBB	Incomplete right bundle branch block
		ILBBB	Incomplete left bundle branch block
		CLBBB	Complete left bundle branch block
		CRBBB	Complete right bundle branch block
		_AVB	AV block
		IVCB	Non-specific intraventricular conduction disturbance (block)
		WPW	Wolff–Parkinson–White syndrome
HYP	Hypertrophy	LVH	Left ventricular hypertrophy
		RHV	Right ventricular hypertrophy
		LAO/LAE	Left atrial overload/enlargement
		RAO/RAE	Right atrial overload/enlargement
		SEHYP	Septal hypertrophy
MI	Myocardial Infarction	AMI	Anterior myocardial infarction
		IMI	Inferior myocardial infarction
		LMI	Lateral myocardial infarction
		PMI	Posterior myocardial infarction
STTC	ST/T change	ISCA	Ischemic in anterior leads
		ISCI	Ischemic in inferior leads
		ISC_	Non-specific ischemic
		STTC	ST-T changes
		NST_	Non-specific ST changes

**Table 4:** Data augmentation techniques and the corresponding description.

Technique	Description
Adding noise	Adding noise of Gaussian distribution with a mean of 0 and a standard deviation of 0.01.
Amplification	Multiply by a gain k = 1 + x, x ∈ [0.001, 0.01].
Attenuation	Multiply by a gain k = 1 − x, x ∈ [0.001, 0.01].

**Table 5:** The CNN model parameters.

Parameter	Value
Convolutional layers for Branch 1 (ECG signals)	32, 64, 128, 256, 512
Convolutional layer size for Branch 1	3 × 1
Max pooling layer size	2 × 1
Dense layers for Branch 1	100, 32
Dense layers for Branch 2 (demographic data)	100, 64, 32, 16
Drop out size for Branch 1	0.4
Drop out size for Branch 2	0.4
Dense layers for concatenated branch	10, 10, num_classes (5, 10, 15)
Drop out size for concatenated branch	0.2
Convolutional layer activation function	ReLU
Output activation function	Softmax
Loss function	Crossentropy
Optimizer	Adam (Learning rate = 0.001)
Callbacks	EarlyStopping, Reduce LearningRate, Save CheckPoint
Max_Epochs	60
Batch size	16

**Table 6:** Architectural and training parameters of the proposed VGG-based model.

Parameter	Value
Convolutional layers for Branch 1 (ECG signals)	64, 64, 128, 128, 256, 256, 512, 512
Convolutional layer size for Branch 1	3 × 1
Max pooling layer size	2 × 1
Dense layers for Branch 1	512, 512
Dense layers for Branch 2 (demographic data)	100, 64, 32, 16
Drop out size for Branch 1	0.5
Drop out size for Branch 2	0.4
Dense layers for concatenated branch	10, 10, num_classes (5, 10, 15)
Drop out size for concatenated branch	0.2
Convolutional layer activation function	ReLU
Output activation function	Softmax
Loss function	Crossentropy
Optimizer	Adam (Learning rate = 0.001)
Callbacks	EarlyStopping, Reduce LearningRate, Save CheckPoint
Max_Epochs	60
Batch size	16

**Table 7:** Components of a binary confusion matrix showing TP, TN, FP, and FN.

Actual/Predicted	Predicted Positive	Predicted Negative
Actual Positive	True Positive (TP)	False Negative (FN)
Actual Negative	False Positive (FP)	True Negative (TN)

**Table 8:** Binary classification (normal vs. one disease from the remaining four superclasses) with and without patient data using the CNN model for the test set.

	Without Patient Data				With Patient Data			
	NORM and MI	NORM and STTC	NORM and CD	NORM and HYP	NORM and MI	NORM and STTC	NORM and CD	NORM and HYP
**Precision**	0.9652	0.9397	0.9195	0.9752	0.9745	0.9410	0.9244	0.9770
**Recall**	0.9652	0.9397	0.9195	0.9752	0.9745	0.9410	0.9244	0.9770
**F1_score**	0.9279	0.9114	0.8690	0.8765	0.9321	0.9132	0.8794	0.8995
**Accuracy**	0.9652	0.9397	0.9195	0.9752	0.9745	0.9410	0.9244	0.9770
**Training time (s)**	260	252	223	179	289	338	309	306
**Average accuracy**	0.9499				0.9542			

**Table 9:** Binary classification (normal vs. one disease from the remaining 9 subclasses) results without patient data using the CNN model for the test set.

	NORM and STTC	NORM and AMI	NORM and IMI	NORM and LAFB/ LPFB	NORM and LVH	NORM and IRBBB	NORM and CLBBB	NORM and ISCA	NORM and CRBBB
**Precision**	0.9243	0.9838	0.9678	0.9687	0.9855	0.9627	0.9965	0.9925	0.9987
**Recall**	0.9243	0.9838	0.9678	0.9687	0.9855	0.9627	0.9965	0.9925	0.9987
**F1_score**	0.8456	0.9641	0.8832	0.8990	0.9440	0.8435	0.9538	0.9050	0.9907
**Accuracy**	0.9243	0.9838	0.9678	0.9687	0.9855	0.9627	0.9965	0.9925	0.9987
**Training time (s)**	242	227	197	198	176	181	206	184	241
**Average accuracy**	**0.9756**								

**Table 10:** Binary classification (normal vs. one disease from the remaining 9 subclasses) results with patient data using the CNN model for the test set.

	NORM and STTC	NORM and AMI	NORM and IMI	NORM and LAFB/LPFB	NORM and LVH	NORM and IRBBB	NORM and CLBBB	NORM and ISCA	NORM and CRBBB
**Precision**	0.9255	0.9850	0.9747	0.9744	0.9797	0.9708	0.9986	0.9932	0.9986
**Recall**	0.9255	0.9850	0.9747	0.9744	0.9797	0.9708	0.9986	0.9932	0.9986
**F1_score**	0.8609	0.9647	0.9092	0.9168	0.9227	0.8621	0.9739	0.9122	0.9739
**Accuracy**	0.9255	0.9850	0.9747	0.9744	0.9797	0.9708	0.9986	0.9932	0.9986
**Training time (s)**	311	283	224	289	219	276	312	236	310
**Average accuracy**	0.9778								

**Table 11:** Binary classification (normal vs. abnormal) results without/with patient data using the CNN model for the test set.

	Without Patient Data	With Patient Data
**Precision**	0.8821	0.8934
**Recall**	0.8821	0.8934
**F1_score**	0.8808	0.8929
**Accuracy**	0.8821	0.8934
**Training time (s)**	325	495

**Table 12:** Multiclass classification results with/without patient data using the CNN model for the test set.

Metric	Without Patient Data			With Patient Data		
	5 Superclasses	10 Subclasses	15 Subclasses	5 Superclasses	10 Subclasses	15 Subclasses
**Precision**	0.7986	0.8145	0.7913	0.8270	0.8341	0.8131
**Recall**	0.7895	0.7613	0.7141	0.7659	0.7523	0.6613
**F1_score**	0.6804	0.6469	0.4272	0.6757	0.6261	0.3889
**Accuracy**	0.7949	0.7835	0.7370	0.7955	0.7960	0.7261
**Training time (s)**	340	304	338	666	626	571

**Table 13:** Binary classification (normal versus one disease from the remaining 4 superclasses) using the VGG16 model for the test set.

Metric	With Patient Data			
	NORM and MI	NORM and STTC	NORM and CD	NORM and HYP
Precision	0.9564	0.9202	0.9297	0.9752
Recall	0.9564	0.9202	0.9297	0.9752
F1_score	0.9306	0.8771	0.8916	0.8823
Accuracy	0.9564	0.9202	0.9297	0.9752
Training time (s)	398	459	460	446
Average accuracy	**0.9454**			

**Table 14:** Binary classification (normal versus one disease from the remaining 9 subclasses) using the VGG16 model for the test set.

Metric	NORM and STTC	NORM and AMI	NORM and IMI	NORM and LAFB/ LPFB	NORM and LVH	NORM and IRBBB	NORM and CLBBB	NORM and ISCA	NORM and CRBBB
**Precision**	0.9390	0.9805	0.9731	0.9695	0.9787	0.9634	0.9974	0.9631	0.9994
**Recall**	0.9390	0.9805	0.9731	0.9695	0.9787	0.9634	0.9974	0.9631	0.9994
**F1_score**	0.8724	0.9533	0.9014	0.8952	0.9136	0.8677	0.9650	0.8952	0.9964
**Accuracy**	0.9390	0.9805	0.9731	0.9695	0.9787	0.9634	0.9974	0.9631	0.9994
**Training time (s)**	408	394	608	371	484	486	314	517	785
**Average accuracy**	**0.9738**								

**Table 15:** Binary classification (normal and abnormal) results using the VGG16 model for the test set.

Precision	Recall	F1_score	Accuracy	Training time (s)
0.8729	0.8729	0.8701	0.8729	557

**Table 16:** Multiclass classification results using the VGG16 model for the test set.

Metric	5 Superclasses	10 Subclasses	15 Subclasses
**Precision**	0.7685	0.7884	0.7179
**Recall**	0.7628	0.7515	0.6855
**F1_score**	0.7656	0.7695	0.7013
**Accuracy**	0.7653	0.7685	0.6929
**Training time (s)**	1024	1005	1001

**Table 17:** The proposed models’ performance against some recently published works.

Ref.	Year	Model	Average Accuracy					
			Binary			Multiclass		
			Normal and Abnormal	Normal vs. One Superclass	Normal vs. One Subclass	5 Classes	10 Classes	15 Classes
[27]	2022	CNN-LSTM		90.94%		74.33%		
[31]	2024	GRU		80.69%				
[32]	2021	QRS entropy+ Raw signal	89.8%			75.8%		
[44]	2022	FSL + XGBoost	88.9%			75.2%		
[54]	2021	CNN + Entropy features.	89.2%			76.5%		
[62]	2023	CNN				70.11%		
Proposed	2025	VGG (Incorporating patient data)	87.29%	94.54%	97.38%	76.53%	76.85%	69.29%
Proposed	2025	CNN (Incorporating patient data)	**89.34%**	**95.42%**	**97.78%**	**79.55%**	**79.60%**	**72.61%**

**Table 18:** Binary classification (normal vs. one disease from the remaining 4 superclasses) for the test set using the CNN model trained on the augmented ECG dataset.

Metric	NORM and MI	NORM and STTC	NORM and CD	NORM and HYP
**Precision**	0.9499	0.9246	0.9251	0.9714
**Recall**	0.9499	0.9246	0.9251	0.9714
**F1_score**	0.9175	0.8977	0.8874	0.8846
**Accuracy**	0.9499	0.9246	0.9251	0.9714
**Training time (s)**	294	235	257	274
**Average accuracy**	0.9428			

**Table 19:** Binary classification (normal vs. one disease from the 9 subclasses) for the test set using the CNN model trained on the augmented ECG dataset.

Metric	NORM and STTC	NORM and AMI	NORM and IMI	NORM and LAFB/LPFB	NORM and LVH	NORM and IRBBB	NORM and CLBBB	NORM and ISCA	NORM and CRBBB
**Precision**	0.9267	0.9883	0.9725	0.9711	0.9763	0.9641	1.0000	0.9926	1.0000
**Recall**	0.9267	0.9883	0.9725	0.9711	0.9763	0.9641	1.0000	0.9926	1.0000
**F1_score**	0.8669	0.9728	0.9035	0.8972	0.9159	0.8693	0.9839	0.9060	1.0000
**Accuracy**	0.9267	0.9883	0.9725	0.9711	0.9763	0.9641	1.0000	0.9926	1.0000
**Training time (s)**	277	273	278	294	266	297	381	276	288
**Average accuracy**	0.9768								

**Table 20:** Binary classification (normal vs. abnormal) for the test set using the CNN model trained on the augmented ECG dataset.

Precision	Recall	F1_score	Accuracy	Training time (s)
0.8893	0.8893	0.8820	0.8893	322

**Table 21:** Multiclass classification results for the test set using the CNN model trained on the augmented ECG dataset.

Metric	5 Superclasses	10 Subclasses	15 Subclasses
**Precision**	0.7732	0.7906	0.7220
**Recall**	0.7666	0.7867	0.7144
**F1_score**	0.6460	0.6499	0.4370
**Accuracy**	0.7689	0.7889	0.7147
**Training time (s)**	631	1294	1868

**Table 22:** CNN accuracy results with and without data augmentation for the test set.

Dataset	Binary Classification			Multiclass Classification		
	Binary (Normal vs. Abnormal)	Normal vs. One of 5-Super Classes	Normal vs. One of 10-Subclasses	5 Classes	10 Classes	15 Classes
**PTB- XL dataset without augmentation**	0.8934	0.9542	0.9778	0.7955	0.7960	0.7261
**PTB- XL dataset with augmentation**	0.8893	0.9428	0.9768	0.7689	0.7889	0.7147

## Data Availability

The dataset used in this work is publicly available at: https://physionet.org/content/ptb-xl/1.0.3/records100/21000/ (accessed on 21 June 2025).
